# Thyroglobulin level at week 16 of pregnancy is superior to urinary iodine concentration in revealing preconceptual and first trimester iodine supply

**DOI:** 10.1111/mcn.12470

**Published:** 2017-06-07

**Authors:** Monika Katko, Andrea Anett Gazso, Ildiko Hircsu, Harjit Pal Bhattoa, Zsuzsanna Molnar, Bela Kovacs, David Andrasi, Janos Aranyosi, Rita Makai, Lajos Veress, Olga Torok, Miklos Bodor, Laszlo Samson, Endre V. Nagy

**Affiliations:** ^1^ Division of Endocrinology, Department of Medicine, Faculty of Medicine University of Debrecen Debrecen Hungary; ^2^ Department of Laboratory Medicine, Faculty of Medicine University of Debrecen Debrecen Hungary; ^3^ Institute of Food Science University of Debrecen Debrecen Hungary; ^4^ Kenézy Gyula Hospital Debrecen Hungary; ^5^ Department of Obstetrics and Gynecology, Faculty of Medicine University of Debrecen Debrecen Hungary

**Keywords:** iodine status, pregnancy, thyroglobulin, thyroid

## Abstract

Pregnant women are prone to iodine deficiency due to the increased need for iodine during gestation. Progress has recently occurred in establishing serum thyroglobulin (Tg) as an iodine status biomarker, but there is no accepted reference range for iodine sufficiency during pregnancy. An observational study was conducted in 164 pregnant women. At week 16 of gestation urinary iodine concentration (UIC), serum Tg, and thyroid functions were measured, and information on the type of iodine supplementation and smoking were recorded. The parameters of those who started iodine supplementation (≥150 μg/day) at least 4 weeks before pregnancy (*n* = 27), who started at the detection of pregnancy (*n* = 51), and who had no iodine supplementation (*n* = 74) were compared. Sufficient iodine supply was found in the studied population based on median UIC (162 μg/L). Iodine supplementation ≥150 μg/day resulted in higher median UIC regardless of its duration (nonusers: 130 μg/L vs. prepregnancy iodine starters: 240 μg/L, and pregnancy iodine starters: 205 μg/L, *p* < .001, and *p* = .023, respectively). Median Tg value of pregnancy starters was identical to that of nonusers (14.5 vs. 14.6 μg/L), whereas prepregnancy starters had lower median Tg (9.1 μg/L, *p* = .018). Serum Tg concentration at week 16 of pregnancy showed negative relationship (*p* = .010) with duration of iodine supplementation and positive relationship (*p* = .008) with smoking, a known interfering factor of iodine metabolism, by multiple regression analysis. Serum Tg at week 16 of pregnancy may be a promising biomarker of preconceptual and first trimester maternal iodine status, the critical early phase of foetal brain development.

## 1 INTRODUCTION

In spite of continuous efforts mild iodine deficiency remains an unresolved issue in many parts of the world (Lee, Cho, Shin, & Song, 2016; Völzke et al., 2016). Measurement of median urinary iodine concentration (UIC) is the recommended method to define iodine status at the population level (World Health Organization, 2007). Due to its large day‐to‐day variation, UIC, especially from spot urine samples, is not appropriate for assessing iodine status in individual cases (König, Andersson, Hotz, Aeberli, & Zimmermann, 2011; Soldin, 2002); however, its application at the population level is supported by well‐established criteria in various groups (World Health Organization, 2007). UIC reflects the recent iodine intake of the last 24–48 hr and carries limited information on individual existing iodine stores (World Health Organization, 2007). Thyroglobulin (Tg), the high molecular weight precursor of thyroid hormone synthesis, has been demonstrated to be a suitable marker of thyroid iodine economy of a longer time due to its strong association with existing iodine stores in euthyroid individuals (Bílek, Čeřovská, & Zamrazil, 2015; Knudsen et al., 2001; Ma & Skeaff, 2014; Rasmussen et al., 2002; Vejbjerg et al., 2009b). During the synthesis of thyroid hormones, coupled iodinated tyrosine residues in the Tg molecule form triiodothyronine (T3) and thyroxine (T4) and are subsequently released by proteolysis, whereas a small amount of Tg also appears in the bloodstream (de Vijlder, Ris‐Stalpers, & Vulsma, 1999; Herzog, 1983). Serum Tg concentration increases in iodine deficiency (Bílek et al., 2015; Knudsen et al., 2001; Rasmussen et al., 2002; Vejbjerg et al., 2009b). Intervention studies reveal that decrease of Tg concentration in response to iodine supplementation is an indicator of the improvement in iodine status (Benmiloud et al., 1994; Ma, Venn, Manning, Cameron, & Skeaff, 2016; Missler, Gutekunst, & Wood, 1994).

Pregnant women and their foetuses are highly sensitive to iodine deficiency thereby giving a high priority to the monitoring of iodine status at these life stages (Moog et al., 2017; Zimmermann, 2009). First trimester of pregnancy is crucial from the point of maternal thyroid hormone dependent foetal brain development (Bernal & Nunez, 1995; Moog et al., 2017). During pregnancy, a median UIC of 150–249 μg/L indicates iodine sufficiency (World Health Organization, 2007). UIC normalized to urine creatinine concentration (μg iodine/g creatinine; UIC/Cr) was found to be a better indicator of iodine status during pregnancy (Li et al., 2016), as well as during breastfeeding (Andersen, Møller, & Laurberg, 2014), due to reduced variations caused by differences in urine volume.

Up till now, serum Tg measurement has not been widely used in assessing iodine status during pregnancy. In addition to iodine supply, thyroid function and thyroid size are also major determinants of the serum Tg concentrations in nonpregnant adults (Knudsen et al., 2001). It is not entirely clear how and to what extent the combined effect of the gestational hormonal changes and the increased demand for iodine is reflected in Tg concentrations. During pregnancy, the secretory activity of the thyroid gland increases to meet the higher demand for thyroid hormones (Moleti, Trimarchi, & Vermiglio, 2014); this reaches a plateau by week 16. Ending by the end of the first trimester, high human chorionic gonadotropin (hCG) concentration has a transient thyrotropic effect (Hershman, 2004), which may further interfere with the causal relationship between iodine intake and serum Tg concentration. The hCG concentration was a significant predictor for Tg in a group of iodine deficient pregnant women (Koukkou, Ilias, Mamalis, Adonakis, & Markou, 2016). Although recent data suggest that a median Tg below 10 μg/L (with less than 3% of individual values ≥44 μg/L) indicates iodine sufficiency in pregnant women using a dried blood spot Tg assay (Stinca et al., 2017), no accepted serum Tg reference range has been established for maternal iodine sufficiency.

We assumed that in pregnant women living in iodine deficient area serum Tg at week 16 of gestation may carry information on iodine intake in the first trimester, the critical phase of foetal brain development. We aimed to verify if pregestational initiation of iodine supplementation is more advantageous in comparison to supplementation start at the time of pregnancy detection using serum Tg as a biomarker.

**Key messages**

- Unlike urinary iodine concentration, serum thyroglobulin (Tg) concentrations at week 16 of gestation reflects both prepregnancy and first trimester iodine status covering the critical period of foetal brain development.
- Using serum Tg as a biomarker of iodine status, our data verify that women of child‐bearing age living in iodine deficient areas should start iodine supplementation before pregnancy to maintain optimal iodine supply to the thyroid during pregnancy.
- Weak or missing correlation between urinary iodine concentration and serum Tg during pregnancy can be the result of recent lifestyle changes (starting iodine containing supplements and cessation of smoking) associated with the detection of pregnancy.

## 2 SUBJECTS AND METHODS

### 2.1 Subjects

The study was conducted in Debrecen, the second largest city of Hungary, in 2014. One hundred and eighty‐nine healthy pregnant women visiting either the Clinical Center of the University of Debrecen or Kenézy Gyula Hospital for routine pregnancy control, without a personal history of thyroid disease, were invited to participate in the study at week 16 of gestation. The study protocol was approved by the Institutional Ethics Committee of the University of Debrecen. After informed consent was given, morning nonfasting spot urine and blood samples were collected.

### 2.2 Laboratory measurements

After separation, serum samples were stored at −70 °C and urine samples were stored at −20 °C until analysis.

UIC was measured by inductively coupled plasma mass spectrometry. Urine samples were diluted 10 times with 0.5% NH_3_ solution before the analysis. Tellurium was used as internal standard (20 μg/L). The diluted samples were analysed by a Thermo Scientific XSeries 2 ICP‐MS (Thermo Fisher Scientific Inc., Bremen, Germany) with a hexapole collision/reaction cell. Intra‐assay coefficients of variation (CV) were 2–5% and inter‐assay CV values were 10–15%.

Women were classified as smokers according to the concentration of the nicotine metabolite, cotinine in their urine, measured by gas chromatography–mass spectrometry as described by da Cunha et al. (2013). A Thermo Scientific Finnigan Trace GC Ultra gas chromatograph with Finnigan Polaris Q mass spectrometer (Thermo Fisher Scientific Inc., Bremen, Germany) was used. The cutoff value of urine cotinine concentration for nicotine exposure was 100 ng/mL. Intra‐assay CV values were 1–3% and inter‐assay CV values were 4–5%.

Urine creatinine concentration was measured immediately after collection by Jaffé's method using a Cobas 6000 analyser (Roche Diagnostics GmbH, Mannheim, Germany). Normal range was 0.29–2.26 g/L. Intra‐assay and inter‐assay CV values were 1–2% and 2–4%, respectively.

Serum Tg concentration was measured by chemiluminescent immunoassay (LIAISON^®^‐Tg, DiaSorin S.p.A., Saluggia, Italy). The test was calibrated using the reference standard CRM‐457. Nonpregnant adult reference range was 0.2–70 ng/mL. Intra‐assay CV values were 3–6%, and inter‐assay CV values were 6–11%. Concentrations of anti‐Tg antibodies (TgAb) were measured by radioimmunoassay with DYNOtest anti‐Tg (BRAHMS Diagnostica GmbH, Hennigsdorf, Germany) on a Ria‐mat 280 analyser (Stratec, Birkenfeld, Germany). Intra‐assay CV values were 2–8% and inter‐assay CV values were 3–8%. Women with TgAb concentration above 60 IU/L were considered TgAb positive.

Total hCG concentration was measured using enzyme‐linked immunosorbent assay technique. The normal median value at week 16 of pregnancy in our laboratory is 28110 IU/L. Intra‐assay CV value was 3.6%, and inter‐assay CV value was 5.9%.

Serum thyroid‐stimulating hormone (TSH), free T4 (fT4), free T3 (fT3) concentrations were measured using electrochemiluminescence immunoassay on Modular Analytics E170 analyser (Roche Diagnostics GmbH, Mannheim, Germany). Reference ranges were 0.3–4.2 mU/L for TSH, 12–22 pmol/L for fT4 and 2.4–6.3 pmol/L for fT3. Intra‐assay CV values were 1–2%, 2–5%, and 1–3%, inter‐assay CV values were 2–3%, 2–7%, and 2–8%, respectively.

### 2.3 Questionnaire

All subjects completed a questionnaire covering information on age, current medications if any, and iodine supplementation: yes‐no questions about regular use of iodized salt before and during current pregnancy, regular use of pregnancy supplement or other dietary supplement before and during current pregnancy with product names and duration of use. Smoking habits were also recorded (never smoked, quit smoking before conception, and quit smoking when pregnancy was detected or current smoker).

### 2.4 Statistical analysis

Statistical analysis was performed by STATISTICA 12 software (Statsoft Inc. Tulsa, OK, USA). The distribution of continuous variables was checked by the Kolmogorov–Smirnov test. For analysis of the relationship between nonnormal distributed continuous variables Spearman's rank‐order correlation test was performed. To compare continuous variables between two or more subgroups, for normal distributed data Student's *t* test or one‐way analysis of variance was applied, whereas for nonnormal distributed data Mann Whitney‐U test or Kruskal–Wallis H test was used as appropriate. Results were expressed as mean ± standard deviation in case of normal distribution, or median and 25th and 75th percentiles (interquartile range, IQR) in case of nonnormal distribution. The stochastic relationships of discrete variables were analysed by chi‐square test. Multiple linear regression analysis was performed using logarithmically transformed serum Tg (logTg) as a dependent variable and logTSH, loghCG, duration of iodine supplementation (≥150 μg/day; coded as 0 = nonusers, 1 = pregnancy iodine starters, and 2 = prepregnancy iodine starters) and smoking status (coded as 0 = never smoked, 1 = former smokers, and 2 = current smokers) as potential predictor variables. *p* values below .05 were considered statistically significant.

## 3 RESULTS

Twenty‐five (13%) of 189 women had elevated (>60 IU/L) TgAb concentration. Because endogenous TgAb is known to interfere with immunological methods for measurement of Tg (Iervasi, Iervasi, Carpi, & Zucchelli, 2006), women with positive TgAb were excluded from further analysis. Characteristics of the TgAb negative subjects (study subjects, *n* = 164) are shown in Table 1.

**Table 1:** Characteristics of the TgAb negative study population (*n* = 164)

Parameters	Values	
Age (years)	30.2 ± 5.2	
UIC (μg/L)	162 (92–290)	
UIC/Cr (μg/g creatinine)	148 (94–254)	
Tg (μg/L)	14.2 (8.5–24.7)	
Tg ≥44 μg/L (*n*)	14 (9%)	
TSH (mU/L)	1.8 (1.2–2.4)	
fT4 (pmol/L)	13.3 (12.5–14.4)	
fT3 (pmol/L)	4.8 (4.4–5.0)	
hCG (IU/L)*	30921 (21926–43008)	
Smoking status (verified by urine cotinine levels)		
Never smoked (*n*)	81 (49.4%)	
Former smoker (*n*)	40 (24.4%)	
Current smoker (*n*)	43 (26.2%)	
Iodine supplementation (self‐reported)	preconceptual	current
None (*n*)	71 (43.3%)	48 (29.3%)
Only iodized salt (*n*)	57 (34.8%)	30 (18.3%)
Only iodine containing pregnancy supplement (*n*)	14 (8.5%)	46 (28.0%)
Both iodized salt and iodine containing pregnancy supplement (*n*)	18 (11.0%)	36 (22.0%)
Not known (*n*)	4 (2.4%)	4 (2.4%)

*Note*. Age is expressed as mean ± standard deviation; laboratory parameters are expressed as median and interquartile range (in parenthesis). UIC = urinary iodine concentration; UIC/Cr = creatinine‐normalized urinary iodine concentration; Tg = thyroglobulin; TSH = thyroid‐stimulating hormone; fT4 = free thyroxine; fT3 = free triiodothyronine; hCG = human chorionic gonadotropin.

* *n* = 159.

Significant positive correlation was found between fT4 and fT3 (r_s_ = 0.333, *p* < .001). Neither TSH nor hCG was associated with any other studied parameter. No correlations between serum Tg concentrations and TSH, fT3, fT4, or hCG concentrations were found. There was only a weak negative correlation between serum Tg concentrations and UIC/Cr (r_s_ = −0.164, *p* = .035).

### 3.1 Iodine supplementation

Less than half of the women used iodized salt; their number did not differ significantly before pregnancy (46%) and during pregnancy (40%; Table 1). At the time of the study, all pregnancy supplements were available over the counter in Hungary, and all, but one pregnancy supplement contained iodine. Fifty‐five women (35%) reported regular use of a pregnancy supplement before pregnancy, and 32 of them (20% of all participants) were taking iodine‐containing pregnancy supplements. Supplement use become more frequent after the detection of pregnancy: 83% versus 35%, *p* < .001 for all supplements, and 50% versus 20%, *p* < .001 for iodine containing supplements.

**Figure 1:**
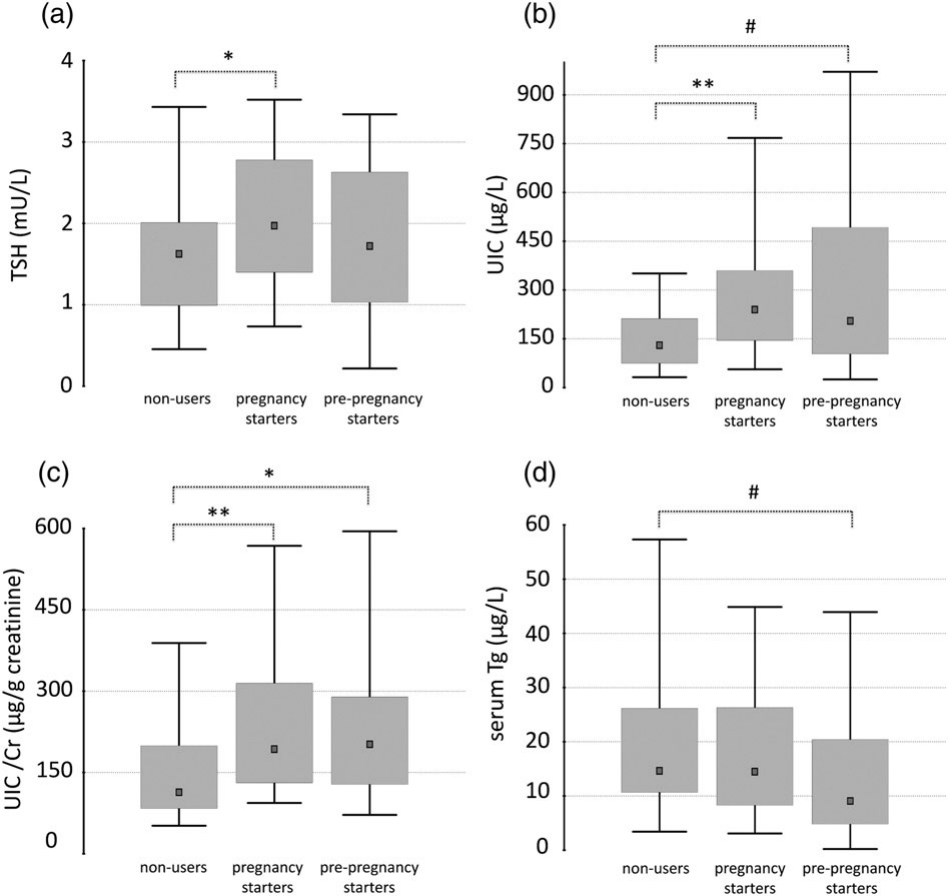
The effect of the duration of ≥150 μg/day iodine containing pregnancy supplement consumption on (a) thyroid‐stimulating hormone (TSH) concentrations, (b) urinary iodine concentration (UIC), (c) creatinine‐normalized UIC (UIC/Cr), and (d) serum thyroglobulin (Tg) concentrations. Pregnancy starters: women who started iodine supplementation at the time of detection of pregnancy; prepregnancy starters: women who started iodine supplementation at least 4 weeks before pregnancy. Median, interquartile range, and the 5–95% ranges are shown. Results of the post hoc tests after Kruskal Wallis H test: ^#^ *p* = .020; ^*^ *p* < .005; ^**^ *p* < .001

First, the impact of the self‐reported iodine supplementation at inclusion (week 16 of pregnancy) was examined on the studied parameters. Women with unknown status of current iodine supplementation (*n* = 4) were excluded from this analysis. Of the 160 women, 78, 2, 56, 17, and 7 reported using 0, 75, 150, 200, and 220 μg daily iodine, respectively. In Table 2, results are shown based on the iodine content of the actual pregnancy supplement. The current consumption of iodized salt did not influence Tg, UIC or UIC/Cr concentrations (data not shown). Unlike in the ≥150 μg iodine/day group, the UIC of the <150 μg iodine/day group failed to reach iodine sufficiency (lower limit of sufficient median UIC in pregnancy by WHO definition is 150 μg/L). Serum Tg showed mild iodine deficiency (using cutoff value: 10 μg/L from Stinca et al., 2017) in both groups (Table 2). The TSH concentrations were higher in the group with ≥150 μg/day iodine intake. Next, we aimed at testing the effect of prepregnancy ≥150 μg daily iodine supplementation. Women were stratified according to the reported duration of iodine containing pregnancy supplement use: nonusers, pregnancy starters (supplementation started at the time of detection of pregnancy), and prepregnancy starters (supplementation started at least 4 weeks before pregnancy; Table 3). Women with unknown status of iodine supplementation before or during pregnancy (*n* = 8), and women who had taken iodine containing supplements before pregnancy and switched to pregnancy supplements without iodine at the time of detection of pregnancy (*n* = 4) were excluded from this part of the analysis. The median durations of supplement use were 10 and 25 weeks in pregnancy starters and prepregnancy starters, respectively. Iodine supplementation ≥150 μg/day, resulted in higher UIC and UIC/Cr compared to nonusers, regardless of its duration. However, prepregnancy starters had lower serum Tg concentrations than pregnancy starters, and the Tg concentrations of pregnancy starters were identical to those of nonusers. Only the group of prepregnancy starters had median serum Tg value below 10 μg/L. TSH was slightly increased in pregnancy starters (Table 3). The results of the post hoc tests are shown in Figure 1.

**Table 2:** Pregnant women are stratified according to the iodine supplementation at inclusion (week 16 of pregnancy)

	<150 μg iodine/day (*n* = 80)	≥150 μg iodine/day (*n* = 80)	*p* value^a^
Age (years)	30.1 ± 5.7	30.4 ± 4.8	.708
UIC (μg/L)	128 (71–210)	239 (141–365)	<.001
UIC/Cr (μg/g creatinine)	115 (83–197)	197 (133–297)	<.001
Tg (μg/L)	14.7 (10.5–24.7)	12.2 (6.9–24.9)	.070
Tg ≥44 μg/L (*n*)	6 (8%)	7 (9%)	.772
TSH (mU/L)	1.66 (1.03–2.01)	1.89 (1.40–2.68)	.003
fT4 (pmol/L)	13.3 (12.4–14.3)	13.3 (12.5–14.3)	.914
fT3 (pmol/L)	4.8 (4.5–5.0)	4.7 (4.4–5.0)	.268
hCG (IU/L)	29375 (21645–41603)	33732 (23612–44414)	.406
Smokers (*n*) never, former, current	37, 20, 23 (46%, 25%, 29%)	41, 20, 19 (51%, 25%, 24%)	.746
Iodized salt consumption (*n*)	30 (38%)	36 (45%)	.335

*Note*. Age is expressed as mean ± standard deviation; laboratory parameters are expressed as median and interquartile range (in parenthesis). UIC = urinary iodine concentration; UIC/Cr = creatinine‐normalized urinary iodine concentration; Tg = thyroglobulin; TSH = thyroid‐stimulating hormone; fT4 = free thyroxine; fT3 = free triiodothyronine; hCG = human chorionic gonadotropin.

a *t* test (age), chi‐square test (rate of smoking, iodized salt consumption, and Tg ≥44 μg/L) and Mann–Whitney‐U test (other parameters).

**Table 3:** Results according to the duration of iodine containing (≥150 μg/day) pregnancy supplement consumption

	Nonusers (*n* = 74)	Pregnancy starters (*n* = 51)	Prepregnancy starters (*n* = 27)	*p* value^a^
Start of iodine supplementation	n.a.	At the time of detection of pregnancy	At least 4 weeks before pregnancy	n.a.
Duration (weeks)	0	10 (5–12)	25 (20–33)	<.001
Age (years)	29.8 ± 5.8	30.2 ± 5.3	30.9 ± 4.0	.641
UIC (μg/L)	130 (75–212)	240 (145–360)	205 (104–492)	<.001
UIC/Cr (μg/g creatinine)	113 (84–199)	193 (131–315)	202 (128–289)	<.001
Tg (μg/L)	14.6 (10.7–26.2)	14.5 (8.3–26.3)	9.1 (4.8–20.4)	.022
Tg ≥44 μg/L (*n*)	6 (8%)	5 (10%)	1 (4%)	.634
TSH (mU/L)	1.62 (0.99–2.01)	1.97 (1.4–2.78)	1.72 (1.03–2.63)	.006
fT4 (pmol/L)	13.3 (12.5–14.4)	13.2 (12.5–14.3)	13.9 (12.0–14.7)	.923
fT3 (pmol/L)	4.8 (4.4–5.0)	4.6 (4.3–5.0)	4.8 (4.5–5.1)	.282
hCG (IU/L)	28391 (21645–40760)	33029 (20801–44414)	34856 (27267–43289)	.419
Iodized salt consumption (*n*)	26 (35%)	21 (41%)	14 (52%)	.311
Smokers (*n*)	23 (31%)	14 (28%)	5 (19%)	.458

*Note*. Age is expressed as mean ± standard deviation; laboratory parameters and the duration of iodine supplement use are expressed as median and interquartile range (in parenthesis). UIC = urinary iodine concentration; UIC/Cr = creatinine‐normalized urinary iodine concentration; Tg = thyroglobulin; TSH = thyroid‐stimulating hormone; fT4 = free thyroxine; fT3 = free triiodothyronine; hCG = human chorionic gonadotropin; n.a. = not applicable.

a Analysis of variance (age), chi‐square test (rate of smoking and Tg ≥44 μg/L), and Kruskal–Wallis H test (other parameters).

### 3.2 Smoking status

Smoking status was verified by urine cotinine concentrations. All 22 women who reported current smoking had urine cotinine concentrations above 100 ng/L. Sixty‐six of the 189 women reported that they had quit smoking before pregnancy or when pregnancy was detected; however, 21 of them had a urine cotinine concentration above 100 ng/L and were classified as smokers for further analysis. Median urinary cotinine concentration in smokers was 724 (IQR: 212–1330) ng/L.

To test the effects of tobacco smoking, nonsmokers, including women who never smoked, had quit smoking before conception, or had quit smoking when pregnancy was detected were compared to current smokers. Although there was no difference between smokers and nonsmokers in UIC and UIC/Cr, both the serum Tg concentrations of smokers and the percentage of elevated Tg (≥44 μg/L) were markedly higher in the smokers' group. TSH and hCG concentrations were lower with a slight increase in fT3 compared to nonsmokers (Table 4).

**Table 4:** The effect of smoking on the studied parameters

	Nonsmokers (*n* = 121)	Smokers (*n* = 43)	*p* value^a^
Age (years)	30.4 ± 4.7	29.8 ± 6.6	.495
UIC (μg/L)	161 (92–290)	168 (119–286)	.575
UIC/Cr (μg/g creatinine)	155 (97–250)	125 (90–255)	.307
Tg (μg/L)	12.3 (7.9–21.7)	21.1 (10.2–39.6)	.006
Tg ≥44 μg/L (*n*)	5 (4%)	9 (21%)	<.001
TSH (mU/L)	1.80 (1.35–2.40)	1.44 (0.88–2.10)	.040
fT4 (pmol/L)	13.3 (12.4–14.2)	13.3 (12.6–14.6)	.359
fT3 (pmol/L)	4.7 (4.4–5.0)	4.9 (4.5–5.1)	.045
hCG (IU/L)	34013 (23472–44836)	27548 (18834–34856)	.021

*Note*. Age is expressed as mean ± standard deviation; laboratory parameters are expressed as median and interquartile range (in parenthesis). UIC = urinary iodine concentration; UIC/Cr = creatinine‐normalized urinary iodine concentration; Tg = thyroglobulin; TSH = thyroid‐stimulating hormone; fT4 = free thyroxine; fT3 = free triiodothyronine; hCG = human chorionic gonadotropin.

*t* test (age), chi‐square test (rate of Tg ≥44 μg/L) and Mann–Whitney‐U test (other parameters).

Nonsmokers were further divided into subgroups of “never smoked” and “former smokers”, that is, smokers who have quit smoking. According to the results of the post hoc test, serum Tg concentrations of current smokers were significantly higher compared to the concentrations of those who never smoked (21.1, IQR: 10.2–39.6 vs. 11.7, IQR: 7.2–18.6, *p* = .003), whereas former smokers presented an intermediate median Tg concentration (16.8 IQR: 9.9–25.9). Although smoking habits influence serum Tg level, exclusion of smokers did not change our findings with respect to the role of iodine supplementation (data not shown).

The result of the multiple regression analysis for logTg using logTSH, loghCG, duration of iodine supplementation ≥150 μg/day, and smoking status as predictor variables was F(4,143) = 4.47, *p* = .002, adjusted R^2^ = 0.09. Beta coefficients with 95% confidence intervals were 0.01 [−0.15, 0.16] for logTSH, −0.06 [−0.22, 0.10] for loghCG, −0.21 [−0.37, −0.05] for duration of iodine supplementation and 0.22 [0.06, 0.38] for smoking status. Duration of iodine supplementation (≥150 μg/day) and smoking status were significant predictors of serum Tg concentration (*p* = .010 and *p* = .008, respectively), whereas TSH and hCG levels were not (*p* = .951 and *p* = .495, respectively).

## 4 DISCUSSION

Mild iodine deficiency has been the iodine status of Hungary for decades. Although the use of iodized salt remains voluntary in Hungary and is not backed by iodine deficiency disorders (IDD) prevention and monitoring programs, iodine supplementation during pregnancy in the form of pregnancy multivitamins has become widely accepted. In this study, our aim was to compare UIC, UIC/Cr, and serum Tg as biomarkers of iodine supply in pregnant women in a geographical region where previously iodine deficiency had been found (Mezosi et al., 2000).

Week 16 of pregnancy was chosen for sample collection to avoid the potential thyroid stimulatory effect of the peaking hCG around week 12 (Moleti et al., 2014), to represent the iodine supply at early gestation, which is known to be critical from the point of foetal brain development (Moog et al., 2017), and because the growing demand for thyroid hormones during early pregnancy reaches its plateau by week 16 (Moleti et al., 2014).

We found sufficient iodine supply based on UIC according to the WHO criteria (World Health Organization, 2007) and mild iodine deficiency based on serum Tg using the cutoff values published by Stinca et al. (2017) in pregnant women at week 16 of pregnancy. Further, there was only a weak correlation between Tg and creatinine‐normalized UIC in the studied population. There are several possible explanations for this. Number one, UIC, at least in mild to moderate iodine deficiency, is largely dependent on short term iodine supply, whereas Tg values reflect a longer time period and existing iodine stores. Indeed, when we analysed UIC and serum Tg concentrations according to the duration of iodine supplementation, we found that UIC was more dependent on current iodine supplementation, whereas serum Tg was rather dependent on the duration of iodine supplementation: median Tg concentration of those who started iodine supplementation at the time of pregnancy detection was similar to that of nonusers, whereas their UIC and UIC/Cr were close to that of prepregnancy starters (Figure 1b–d). Second, the actual daily iodine intake may fluctuate due to the diet and the varying compliance in iodine supplementation, which is immediately reflected in the UIC. The household use of iodized salt alone failed to exert any effect on either UIC or Tg concentrations. Our data show that iodine containing pregnancy supplements contribute significantly to the optimum urinary iodine excretion; however, the loading of iodine stores, as reflected in Tg concentrations is only achieved by starting supplementation before pregnancy. This finding reinforces the importance of preconceptual iodine supplementation. According to our results, only iodine supplementation initiated in the pregestational period was sufficient to achieve optimum serum Tg by the week 16 of gestation at the population level. This finding is in line with a previous randomized controlled trial when the authors found that 24 weeks of iodine supplementation (150 μg/day) was needed to observe a fall of median serum Tg below the cut‐off value in iodine deficient adults (Ma et al., 2016).

Serum TSH concentrations of those who started iodine supplementation at the time of detection of pregnancy, but not in prepregnancy starters were significantly higher compared to nonusers. These results are consonant with others who found UIC‐related increment in serum TSH concentrations corresponding to the rising regional iodine intake (Guan et al., 2008) or an increase in serum TSH in relation to the introduction of mandatory salt iodization (Vejbjerg et al., 2009a). We assume that higher TSH concentrations are part of the adaptation process to the recently increased iodine intake. This further confirms the need for iodine supplementation in women of child‐bearing age living in iodine deficient areas to maintain optimal thyroid function, at least until iodine sufficiency is achieved at the population level.

Women who reported themselves as nonsmokers and presented a high cotinine concentration were either trying to hide their inability to cease smoking or, alternately, were passive smokers. For classification and data analysis, the actual cotinine concentration was used because the interference of smoking with iodine metabolism was assumed to be independent of the active or passive smoke intake route.

Tobacco smoking has significant effects on thyroid function (Wiersinga, 2013). Large population‐based studies revealed that smokers had significantly lower serum TSH and higher fT4 and fT3 concentrations than nonsmokers in the general population (Belin, Astor, Powe, & Ladenson, 2004; Jorde & Sundsfjord, 2006). In the Northern Finland Birth Cohort smoking in the second trimester was associated with higher fT3 concentrations, but no differences were found in serum TSH concentrations between smokers and nonsmokers (Männistö et al., 2012). We found significantly lower serum TSH and higher fT3 concentrations in current smokers compared to nonsmokers during pregnancy. Similarly to previous observations (Korevaar et al., 2015), significantly lower median hCG concentration was found in smokers than in nonsmokers. Therefore, in addition to the known effect on iodine utilization due to the competitive inhibition of the sodium‐iodide symporter by thiocyanate (Dohán et al., 2003), smoking leads to hCG concentration reduction. Because hCG stimulates sodium‐iodide symporter expression in cytotrophoblast cells (Arturi et al., 2002), smoking may further reduce iodine transfer across the placenta through decrease in hCG concentration.

Several studies suggest that maternal smoking increases serum and cord blood Tg concentrations during pregnancy (Andersen et al., 2013; Brucker‐Davis et al., 2012; Hiéronimus et al., 2013). In subgroup analysis, we found that Tg concentrations of those who never smoked is lower compared to current smokers. We found that the length of the nonsmoker status before conception is important in this respect. According to our results, smoking status is a major contributor to serum Tg concentration in the second trimester, with no impact on urinary iodine. We believe that cigarette smoke interferes with iodine utilization by the thyroid, whereas iodine absorption and UIC are less influenced.

The simultaneous measurement of serum Tg, in addition to the UIC based assessment, may provide useful information on iodine status during pregnancy. This is in sharp contrast with the recent data of Li et al. (2016) and Koukkou et al. (2016), which revealed inconclusive Tg data on a comparable size cohort of pregnant women. We think that the sampling time is important; whereas our pregnant women group was uniform in this respect, as all samples were collected at week 16 of gestation, the other studies were more heterogeneous in this respect including women between 8 and 36 weeks of pregnancy. This may mean that our conclusions cannot be extended to other pregnancy weeks, as the timing of sample collection at other pregnancy weeks may result in different Tg reference values.

Comparisons of the Tg studies are difficult, primarily due to analytical issues (Iervasi et al., 2006). Heterogeneity of the detected Tg epitopes leads to high inter‐assay variation, which can be partly reduced by assay standardization with the certified Tg reference material we have used, CRM‐457 (Feldt‐Rasmussen et al., 1996). The presence of TgAb in the serum interferes with immunoassays for Tg measurement potentially resulting in false high or false low results (Iervasi et al., 2006; Mariotti et al., 1995). This can be avoided by excluding TgAb positive subjects. However, according to the data of others (Stinca et al., 2017; Vejbjerg et al., 2009b) exclusion of TgAb positive cases did not alter their conclusions on the relationship between iodine nutrition and Tg in a population context. We screened the sera for TgAb and TgAb positive subjects were excluded. However, the inclusion of the TgAb positive cases would not have influenced the results with respect to the effect of iodine supplementation on iodine status biomarkers (data not shown).

In this study, we have shown that Tg is a useful marker of the iodine economy of the thyroid gland at week 16 of pregnancy. According to our findings, Tg concentration carries information on iodine supply of a longer period, which includes the pregestational months, compared with UIC that is rather dependent on recent iodine supply. The effect of smoking, a well‐known factor interfering with thyroid hormone synthesis, is reflected in Tg concentration whereas UIC is less affected. Thus, the information carried by Tg at week 16 of pregnancy may outweigh that of UIC; we suspect that the actual iodine store of the thyroid is a major determinant of Tg, whereas UIC is rather dependent on the iodine intake of the last days. Although we aimed to test iodine supply at the population level, it is highly possible that, unlike UIC, Tg may serve as a marker of the individual iodine supply. To clarify this, further studies are warranted.

Our study has several limitations. Due to the relatively small sample size, certain effects may have been overlooked. Sociodemographic information was not collected and the observational nature of the study did not allow us to reduce the effect of socioeconomic factors. Higher maternal socioeconomic status has been linked to access to better nutrition (Novaković et al., 2014) and health care (Larrañaga et al., 2013) and, in iodine deficient areas, to higher iodine intake (Zimmermann, 2013). Indeed, we found that the prevalence of iodised salt use was higher and the prevalence of smoking was lower in prepregnancy starters compared to pregnancy starters (i.e., prepregnancy starters are likely to be more health conscious); although these differences were not significant, they could have contributed to the lower serum Tg concentrations in the group of prepregnancy starters.

The use of serum Tg as a biomarker of iodine status with a 10 μg/L cutoff value seems to be appropriate in pregnant women at week 16 of gestation. Starting iodine containing pregnancy supplements and/or cessation of smoking at the time of detection of pregnancy does not result in significant change in serum Tg concentrations by week 16 of pregnancy, when iodine supplementation is already reflected in UIC. Even so, thyroid economy may be better reflected in Tg than UIC in pregnancy; serum Tg concentration at week 16 of pregnancy may carry information on maternal iodine status of the previous months, including the critical period of foetal brain development.

## CONFLICTS OF INTEREST

The authors declare that they have no conflicts of interest.

## CONTRIBUTIONS

EVN designed and supervised the study, wrote the manuscript, and had primary responsibility for final content; MK performed GC–MS measurements, performed statistical analysis, wrote the manuscript, and created graphs; AAG and IH collected samples and data; HPB and ZM performed the thyroid function tests; BK performed and supervised ICP‐MS measurements; DA performed ICP‐MS measurements; JA, RM, and OT enrolled pregnant women in the study and prepared and processed the questionnaires; LV performed hCG measurement; MB and LS processed the data and coordinated the research. All authors read and approved the final manuscript.
